# SentiHealth: creating health-related sentiment lexicon using hybrid approach

**DOI:** 10.1186/s40064-016-2809-x

**Published:** 2016-07-20

**Authors:** Muhammad Zubair Asghar, Shakeel Ahmad, Maria Qasim, Syeda Rabail Zahra, Fazal Masud Kundi

**Affiliations:** Institute of Computing and Information Technology, Gomal University, D.I. Khan, KP Pakistan; Faculty of Computing and Information Technology in Rabigh (FCITR), King Abdul Aziz University (KAU), Jedda, Saudi Arabia

**Keywords:** Health-related, Lexicon, Bootstrapping, Sentiment detection, Sentiment classification, Hybrid approach, Domain specific

## Abstract

The exponential increase in the health-related online reviews has played a pivotal role in the development of sentiment analysis systems for extracting and analyzing user-generated health reviews about a drug or medication. The existing general purpose opinion lexicons, such as SentiWordNet has a limited coverage of health-related terms, creating problems for the development of health-based sentiment analysis applications. In this work, we present a hybrid approach to create health-related domain specific lexicon for the efficient classification and scoring of health-related users’ sentiments. The proposed approach is based on the bootstrapping modal, a dataset of health reviews, and corpus-based sentiment detection and scoring. In each of the iteration, vocabulary of the lexicon is updated automatically from an initial seed cache, irrelevant words are filtered, words are declared as medical or non-medical entries, and finally sentiment class and score is assigned to each of the word. The results obtained demonstrate the efficacy of the proposed technique.

## Background

The Web is a huge repository of facts and opinions available for people around the world about a particular product, service, issue, policy and health-care (Liu 2011). With the rapid increase in health-related social media sites, individuals are now relying on online medical review sites for exchanging their personal health information, experiences and knowledge (Belt et al. 2010). A recent study (Randeree 2010), regarding usage of online health-related content demonstrated that 80 % of the online users have searched health discussion forums and other online resources for health information, such as disease information, medication they take, and the side effects they feel.

Online drug postings have strong impact on patient’s buying decision, specifically when dealing with non-prescription drugs. Patient’s reviews on drugs help medicinal companies to know the pros and cons of their products. This information plays an important role in improving drug design and advancement (Bos et al. 2008). Moreover, it assists individuals to know about the pros and cons of using different medications.

The sentiment lexicons contain sentiment terms with positive or negative polarities and considered as the steering wheel of all of the sentiment analysis applications (Asghar et al. 2015). In such lexicons, each of the positive or negative terms are associated with their corresponding scores. Examples of positive terms include “awesome”, “lovely”, and “gorgeous”, whereas examples of negative terms include; “dirty”, “poor”, “terrible”, and others. The sentiment lexicons play a pivotal role in determining the semantic orientation of health-related user opinions by storing the sentiment-bearing words along with their numeric scores. The sentiment scores indicate positivity and negativity of a given word. The sentiment lexicons can be developed using different techniques, such as manual, boot-strapping, and corpus-oriented (Asghar et al. 2015). The manual technique operates on the selection and annotation of words by a group of human annotators. This technique is time consuming, costly, and error prone. The boot-strapping technique takes an initial input of seed words and extends it over a collection of web resources, such as Thesaurus.com (Abdalla and Teufel 2006). The major limitation of such technique is that most of the domain specific words are not covered by the resulting lexicon. The corpus-based approach can overcome the limitations of the previous two approaches by incorporating sufficient number of domain specific words. It operates in three steps, namely (1) extraction of candidate words from specialized corpus, (2) searching and matching the words in general-purpose sentiment lexicon, and (3) identifying domain-specific words and calculating their revised sentiment scores. The corpus-based approach provides a sufficient coverage of specialized content by modifying the sentiment score of domain dependent words (Demiroz et al. 2012).

However, problem arises when it is required to assign accurate sentiment scores words in particular domain. For example, the word “Heatstroke” has objective sentiment in SentiWordNet (SWN). However, such polarity is incorrect in the health-related domain, e.g., in the review “The heatstroke laid me down and was unable to move about” should have −ive sentiment score. To address such issues, we need to update sentiment score of words by proposing the hybrid approach, which is combination of bootstrapping and corpus-based techniques.

In this work, we explore the viability of creating health-related sentiment lexicon by proposing hybrid approach based on boot-strapping concepts (e.g., seed list creation, lexicon expansion and redundant words filtering), SWN, and corpus-based techniques (e.g., probability-based improved term weighting measures). The proposed technique is motivated from the previous work performed on creating domain specific lexicons for sentiment analysis (Choi and Cardie 2009; Martineau and Finin 2009; Asghar et al. 2014a, b). The previous studies have used the boot-strapping concepts, or corpus-based strategies, such as term weighting, linear programming and information theory concepts over a labeled dataset. However, we propose to combine boot-strapping concepts, namely: seed cache creation, lexicon expansion, concept tagging and filtering; and corpus-based measures: probability-based sentiment prediction and revised term weighting measures for sentiment scoring using set of labeled dataset.

The main objective of creating health-related sentiment lexicon is to develop a machine readable lexical repository for storing drug-related concepts along with their correct sentiment class and score, which can be used for developing health-related sentiment analysis applications. To accomplish this, we propose an initial seed cache of health-related terms, expand it over a set of web repositories, filter irrelevant words, and finally, tag the selected with Unified Modeling Language System (UMLS) concepts. To detect accurate sentiment class of health-related domain specific words, we propose count-based probability measure. We also propose an enhanced weighting scheme for updating the sentiment score of words by using term frequency (tf), inverse document frequency (idf), and count-based probability measure.

The proposed technique assists in developing a resource of health-related words along with their sentiment class and scores. We demonstrate that creation of such repository has a significant contribution in enhancing the efficiency of health-related sentiment classification. The results obtained demonstrate that the final lexicon is comparable to the baseline methods. The proposed method can benefit many health-related sentiment analysis applications, including sentiment summarization and integration (Fabbrizio et al. 2012; Zhang et al. 2012; Das and Bandyopadhyay 2010; Ly et al. 2011). The sufficient coverage of both uni-gram and bi-gram words in health domain demonstrate the effectiveness of proposed lexicon.

Following is the list of contributions.

- Proposes and implements a hybrid system for creating health-related sentiment lexicon.
- Boot-strapping technique is used to expand the initial seed list of health-related words over a set of web repositories, irrelevant words are filtered using co-reference PMI measure and UMLS tags are used to label words as medical or non-medical entries.
- Count-based probability measure is proposed to assign accurate sentiment class to opinion bearing words.
- Enhanced term weighting scheme is proposed and implemented to assign correct sentiment scores to health-related words.
- Demonstrates the effectiveness of the proposed lexicon with respect to comparing methods.

The rest of paper is structured as follows. “Related work” section demonstrates literature review. In “Methods” section, we describe the proposed method. Experiment design is shown in “Experiments” section. The last section concludes the work with a discussion on how it can be expanded in future.

## Related work

There are several studies regarding development of sentiment lexicons. In this section, we focus on some of relevant studies conducted on the creation of sentiment lexicons in general-purpose and domain specific paradigms using boot-strapping and corpus-based strategies.

Most of the sentiment analysis applications including sentiment classification, opinion and feature extraction, spell corrections and review summarization make use of the sentiment lexicons (Kundi et al. 2014a, b, c, d; Asghar et al. 2014a, b; Ahmad et al. 2014; Pang and Lee 2004). The general purpose subjectivity lexicons are useful for the reviews which are not associated with the specific domain. The studies conducted in the recent past have focused on the generation of general purpose lexicons by utilizing the existing web resources, such as online documents, dataset of user feedbacks and different lexicons. In such lexicons range of semantic scores associated with each word is between −1 and +1.

The WordNet (Miller et al. 1990) is a one of the most popular general purpose lexicon used in sentiment analysis applications. In WordNet, the synonyms are grouped together to form “synsets” and the synsets are semantically arranged into nouns, verbs, adverbs and adjectives. Generally, synsets are connected with each other via semantic relations, such as hypernym, hyponymy, meronym and homonym. WordNet is a comprehensive lexical resource for automatic construction of thesauri, interface for NLP to optimize Internet search (Moldovan and Mihalcea 2000).

WordNet-Affect is used to represent affects behind natural language by utilizing the existing information in WordNet. It assists the developers in extracting affects from user’s reviews, which play a pivotal role in building affective-sensitive systems (Strapparava and Valitutti 2004).

SentiSense is concept-based lexicon used in sentiment analysis-related tasks, such as emotion detection and sentiment classification (Albornoz et al. 2012). It tags meanings of emotions with concepts taken from WordNet and assist in resolving the issue of word sense disambiguation. SentiSense uses fourteen emotional categories along with 5496 words and 2190 synsets.

Linguistic Inquiry and Word Count (LIWC) uses a built dictionary of Pennebaker Dictionary of categories to define its search terms. It has 74 sub-dictionaries having words selected by a set of judges. However, additional dictionaries can also be imported (Pennebaker et al. 2001).

Another all-purpose lexicon is SWN, a publically available resource, with more than sixty thousand synsets obtained dynamically from WordNet (Baccianella et al. 2009). Each word in the SentiWordNet is assigned a positive, negative and neutral scores ranging from 0 to 1 and the sum of these triplets is equal to 1, representing positivity, negativity and neutrality of each word respectively.

General Inquirer (GI) is a general purpose lexicon of English words, annotated manually and divided into different categories, such as “Positive”, “Negative”, “Hostile”, “Power”, “Active” and “Passive” (Stone et al. 1966). It associates different type of information: syntactic, semantic, and pragmatic, to words, tagged with corresponding part-of-speech (POS).

The aforementioned general-purpose sentiment lexicons based on boot-strapping strategies have certain limitations, namely (1) incorrect scoring of domain specific words, and (2) low coverage of domain specific words. For example, the word “relax” has objective polarity in general purpose lexicon, such as SWN, whereas in health-related domain, it has positive polarity. To overcome these limitations, there is growing trend of developing domain-specific sentiment lexicons.

The domain specific lexicons have widely been developed in different domains and languages. Velikovitch et al. (2010) developed a sentiment lexicon from huge collection of web resources using graph propagation technique. Instead of using lexical resources, such SentiWordNet, WordNet and part of speech taggers, they used index terms for evaluating the polarity of words in terms of size and quality. The resulting lexicon contains sufficient number of +ive and −ive words and phrases.

A domain dependent lexicon is proposed by Demiroz et al. (2012) by using term frequency and inverse document frequency weighting mechanism. They changed the polarity of words, which appeared frequently in a particular class (+ive or −ive). For example, if a term has +ive score in SWN, but it has more inclination with −ive class in corpus, then poality of such term is changed accordingly.

Choi and Cardie (2009) integer linear programming approach is suggested to change the polarity of terms on the basis of word and expression level constraints. For example, if a term has −ive score in general-purpose lexicon, but it has more tendency with +ive class in corpus, then poality of such word is modified accordingly.

Goeuriot et al. (2012) used drug reviews’ corpora and different general-purpose lexical resources, such as Subjectivity Lexicon and SentiWordNet for creating health-related domain-specific lexicon. The general purpose lexicon comprises of general opinion words along with their polarity extracted from SentiWordNet. The Information Gain (IG) measure is used to identify the most relevant medical terms. After extraction of the related terms, the polarity is calculated by using the merged and extended lexicon. The accuracy of the proposed method for sentence level polarity classification is computed by using the Vote Flip algorithm. The major problem associated with their approach is the absences of syntactic and linguistic information. Another problem attached to this method is that it neglects the neutral reviews.

Asghar et al. (2015) in their work on creating domain specific lexicon, proposed a unified framework which integrates information theory concepts and revised term weighting measures for predicting and assigning modified scores to domain specific words. They evaluated the system on three datasets: drugs, cars and hotels, and achieved promising results. However, the method can be improved further by incorporating contextual and biomedical features for more efficient classification and scoring of health-related terms.

The aforementioned techniques for creating sentiment lexicons assist in the development of different sentiment analysis applications. However, there is a need to create a domain dependent lexicon for health-related sentiment analysis that can assign accurate sentiment score to a term in health domain because the sentiment score of health-related reviews depends on the specific domain and alters with the change in context (Asghar et al. 2015).

**Fig. 1:**
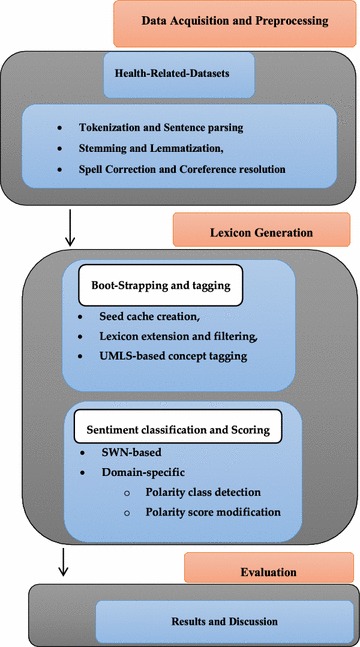
Generic framework of SentiHealth

It gives rise to the development of health-related sentiment lexicon based on drug-related terms and with emphasis on: (1) creating initial seed cache of drug-related terms, (2) extending and filtering the seed entries by using web dictionaries, (3) tagging the extended entries by using Unified Modeling Language Systems (UMLS) to isolate medical and non-medical terms, and (4) sentiment scores are assigned to domain-specific terms by using probability theory and term weighting concepts. The generic framework of the proposed system is shown in Fig. 1.

## Methods

The proposed method for lexicon creation integrates both the boot-strapping and corpus-based approaches for more efficient coverage of health-related content. This consists of following modules: (1) data collection and preprocessing, (2) number of novel algorithms based on boot-strapping concepts such as, seed lists, expansion, filtering, and tagging of lexicon entries, and (3) novel corpus-based algorithms, such as sentiment class detection and sentiment scoring. The data collection and preprocessing module aims at acquiring data from different resources, such as online health forums and publically available datasets; and removing noise from the collected data by applying different preprocessing steps, namely: tokenization, stop word removal, lemmatization, spell correction, and co reference resolution. The proposed method introduces a hybrid approach using an enhanced version of seed lexicon creation and expansion (Asghar et al. 2014a, b), lexicon filtering and tagging (Asghar et al. 2014a, b), SWN-based sentiment scoring (Kundi et al. 2014a, b, c, d), sentiment class detection (Choi and Cardie 2009), and sentiment score modification (Martineau and Finin 2009).

**Fig. 2:**
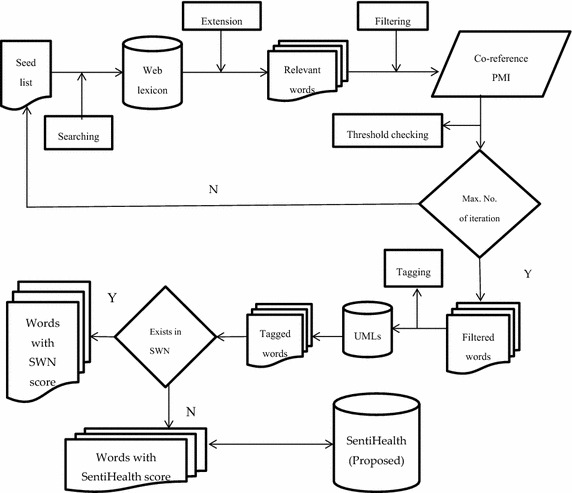
Detailed architecture of SentiHealth

The aim of this work is to enhance the efficiency of the health-related sentiment analysis applications by creating a sentiment lexicon and resolve the issues of low coverage of health-related content in existing lexicons, such as SWN, incorrect sentiment class assignment to domain specific words, and inaccurate scoring of health-related words. The basic idea is to create a seed list of initial words, expand it over set of online repositories, filter the expanded entries by using mathematical measures, tag them with UMLS concepts, and finally, accurate sentiment class and scores are assigned to each entry of the acquired lexicon. The proposed approach works in three steps: (1) firstly, we acquire and preprocess the required data from online sources; (2) secondly, we create an initial seed list, expand it over web resources, filter the irrelevant words, and tag the filtered words with UMLS concepts, and (3) finally, SWN and corpus-based sentiment classification and scoring techniques are applied to classify the words into +ive, −ive or neutral words with appropriate scores. The detailed architecture of the proposed system is won in Fig. 2.

### Data collection and preprocessing

This module deals with acquisition and preprocessing of data acquired from different resources.

#### Data collection

The data acquisition step is used to crawl the webpages from the health-related discussion forums using Beautiful Soup (http://www.crummy.com/software/BeautifulSoup/) a python-based library, used for scrapping the desired webpages. We compiled the dataset from patient’s comments about drugs available on different discussion forums, such as yahoo answers about drugs, Druglib.com, and edrugsearch.com. The users express their reviews about the effectiveness and side effects of a specific drug. Every comment comprises of drug name, quantity of a dose taken, time-period of drug usage, opinions, age of the patient, sex of the patient, efficiency of the drug, side-effects and other conditions. An example review is shown in Table 1.

**Table 1:** Sample patient review on health forum

Drug	Avelox
Reason	Sinus infection
Rating	4
Sex	Female
Age	38
Comments	My major concern is the unusual headache. It’s severe and I am afraid of getting a stroke or aneurism from this. Still getting some bad leg pains, dizziness, crushing fatigue and some stomach upset. It did cure the infection, but these side effects make me unhappy
Dosage	400 mg
Duration	12 days (1 × d)

In addition to the manually compiled dataset, we also used publically available dataset of health reviews available at: http://ir.cs.georgetown.edu/data/adr/. This dataset is comprised of 2500 user reviews for five breast cancer drugs, namely: Anastrozole, Exemestane, Letrozole, Raloxifene, and Tamoxifen. 50 % are used for training and 50 % are used for test dataset. For manually compiled dataset, the sentences are annotated into +ive, −ive or neutral classes using AlchemyAPI (http://www.alchemyapi.com/api) and the classified sentences are stored in the database of SQL Server 2014 to compile the complete dataset. We divide and store the dataset into two separate database files to setup the training and testing corpus. The training corpus consists of 8230 reviews with 44 % +ive, 52 % −ive and 4 % neutral. The testing corpus is comprised of the remaining 17,830 reviews, with 52 % +ive, 38 % −ive and 10 % neutral.

#### Pre-processing

The pre-processing module is used to clean the noisy text by applying different steps, such as tokenization, stop word removal, lemmatization, co-reference resolution, spell correction, and case-conversion.

*Tokenization* The tokenization is the process of splitting the input text into small chunks or pieces, called tokens. We apply tokenization to understand the sentence structure for further text processing. The tokenization can be performed at different levels, such as paragraph level, sentence level and word level. At sentence level, tokenizer splits the text by considering sentence boundary; which represent ending of sentence and starting of the next sentence. At word level, token formulation is performed on the basis of “punctuation marks” or “white spaces”. The tokens may be in the form of “words”, “digits” or “punctuation signs”. In this work, tokenization is performed at word level by using Python code as shown in the algorithm presented below.

*Sentence parsing* We used Stanford parser (Klein and Manning 2003) for assigning Part Of Speech (P.O.S) labels to every word in sentence (Table 2), which assists in getting the sentence structure, typed dependencies, and feature values based on attribute’s mutual dependency. For example, the input sentence: “*The main problem with using Glucophage is severe ankle swelling*”, is assigned P.O.S tags and parsed, as shown in Tables 2 and 3 respectively.

**Table 2:** P.O.S tagging for given sentence

The/DT main/JJ problem/NN with/IN using/VBG Glucophage/NN is/VBZ severe/JJ ankle/NN swelling/VBG

**Table 3:**
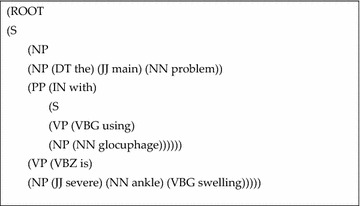
Parsed sentence

*Stop word removal* The stop words are used frequently in natural language. These include ‘is’, ‘to’, ‘for’, ‘an’, ‘are’, ‘in’ and, ‘at’. The stop word elimination plays a pivotal role for dimensionality reduction of the text for further analysis. It assists in the identification of the remaining key words in the natural language becomes easy, and subsequent analysis can be performed efficiently. A list compiled by Savoy (2005), contains vast collection of stop words. The stop word elimination process start with the selection of words and ends by discarding such words from the text. In this work, we propose python-based algorithm for stop words removal process shown as follows:

*Stemming and lemmatization* Stemming and lemmatization are the techniques used for the inflection removal from the text. In stemming, all of the inflected words in the text, are transformed into their base form, namely “stem”. For instance, stemmer converts “books” to “book”, “laughing”, “laughed”, and “laughs” into “laugh”. The stemmer transforms inflected words into their root forms but it is not necessary that every time the converted word is a correct word in dictionary. For example, stemmer converts “manage” to “manag”, “principle” to “princip”, “generated”, “generation” and “generate” to “gener”, which have no existence in English dictionary.

Lemmatization is the process of converting words into their root form or lemma, by maintaining the inflected form (Asghar et al. 2013). For example, the word, “work” is a lemma or base form for the inflected forms “worked”, “working” and “works”. Lemmatization gives more precise results as compared to stemming. For example, lemmas of the words “CARING” and “CARS” are “CARE” and “CAR” respectively, whereas stem for such words is “CAR”, which is incorrect. In this work, stemming is ignored and only lemmatization is applied by using NLTK-based WordNet lemmatizer (http://www.nltk.org/_modules/nltk/stem/wordnet.html).

*Spell correction* Spelling correction is an essential module for a sentiment analysis system, because spelling errors in a text may affect the accuracy of the sentiment classification (Jadhav et al. 2013). There are many causes of miss-spelled words including: typing errors, and deviating from language rules on social media sites and forums. Therefore, spell-checking and correction is incorporated in this work by incorporating spell check plus,^1^ free spell checker^2^ and Jspell^3^ checker in python-based coding.

*Co-reference resolution* The coreference or anaphoric reference resolution is the replacement of anaphoric references with their corresponding antecedent. For example, the text: “*By use of Glucophage I felt stomach pain. It’s severe and also my ankles are swelling badly*.” Contains anaphoric reference. After anaphora resolution, we g,” “*By use of Glucophage I felt stomach pain.* <*Stomach pain*> *is severe and also my ankles are swelling badly*”. We used JavaRAP (Qiu et al. 2004) for coreference resolution, which replaces anaphoric references with their corresponding antecedent and thus anaphora free text is obtained.

### Lexicon generation

The lexicon generation module is comprised of two major components, namely (1) boot-strapping and tagging, and (2) SWN and corpus-based sentiment classification.

#### Boot-strapping and tagging

The boot-strapping component aims at acquiring a large collection of opinion words from a manually compiled seed list of medical terms (HL-1). In first iteration, we expand the seed list over a web lexicon, expanded list is passed through filtering module tod is card the irrelevant entries on the basis of Co-reference PMI measure with user-defined threshold. It results in intermediate lexicon, namely HL-2. In next phase, each of the filtered word is searched in medical lexicon, namely Unified Modelling Language (UMLS) (Bodenreider 2004). The matched words are tagged with the corresponding UMLS-ID.

*Seed cache creation* The initial seed cache is compiled manually, over a set of +ive, −ive, and neutral words, from different publically available online resources, such as MULTUM, NIH, WebMD, MediLexicon, Diabetes.co.uk, and General Inquirer lexicon (Stone et al. 1966). In this work, we adopt a technique proposed by Song et al. (2015) to select seed words by ranking all the words in our datasets (“Data collection” section) according to their frequency count. We manually select five high frequency words distributed over the verb, adverb, noun and adjective categories. The initial seed cache is our first lexicon, named “HL-1”, shown in Table 4.

**Table 4:** Initial seed cache (HL-1)

Type	No. of words		
	Pos	Neg	Neu
Noun	300	300	300
Verb	250	250	250
Adjectives	200	200	200
Adverbs	150	150	150

*Lexicon extension and filtering* In next phase, each term of HL-1 is searched in Web dictionaries, namely, Thesaurus.com.^4^ Goeuriot et al. (2012) in their work on health related sentiment lexicon construction, used Subjectivity Lexicon. However, in contrast to their work, we extend our initial lexicon by using Web Lexicon. We replace subjectivity lexicon with Web Lexicon (WL), as it contains multiple entries like POS, definition, synonyms, and antonyms for a given term. Therefore, it is more beneficial for lexicon expansion, as compared to the subjectivity lexicon. Moreover, the Thesaurus is used to extend the lexicon by including all words within top n entries in treasures.

To stabilize the resulting lexicon, we filter irrelevant terms from the extended lexicon (HL-2, Table 5) by computing the co-reference PMI (Islam and Inkpen 2006) score. This measure assists in finding the semantic relatedness between each of the input term and its corresponding candidate terms. It is computed as follows:

1 $$sim\_score \left( {t1, t2} \right) = \frac{{f^{\alpha } \left( {t1} \right)}}{\alpha 1} + \frac{{f^{\alpha } \left( {t2} \right)}}{\alpha 2}$$

where, *t*1 and *t*2 are the two terms between which semantic relatedness is to be measured. *f*^α^(t1), *f*^α^(t2) represents the summation of all positive PMI scores of whole collection of semantically related terms. α1 and α2 indicates the existence of term *t* in the text and sim_score () function provides a numeric relatedness score between two terms. This score ranges from 0 to 1.

**Table 5:** Partial list of entries from intermediate lexicon (HL_2)

Word	WL repository	Co-reference PMI score
Depression	Downheartedness	1
	Desperation	0.856
	Abasement	0.628
	Discouragement	0.353
	Gloominess	0.335
	Sadness	0.323
	Sorrow	0.3
	Trouble	0.2

In this step, we choose terms with sim_score greater than 0.4; a manually tested threshold. Selected words are included in extended lexicon. Algorithm for lexicon filtering is presented below.

The lexicon extension and filtering experiment is conducted by taking each entry of initial seed cache and performing boot-strap operation to expand it over its synonyms by searching them in web-lexicon (WL). As there could be number of redundant words, we filter them using co-reference PMI score. Resultantly, we get intermediate lexicon, namely HL-2. A partial list of entries of intermediate lexicon is shown in Table 5.

After filtering with threshold of 0.4, we discard the terms with PMI score less than 0.4 and get filtered entries as shown in Table 6.

**Table 6:** Filtered lexicon (HL-2)

Word	WL repository	Co-reference PMI score
Depression	Downheartedness	1
	Desperation	0.856
	Abasement	0.628

*UMLS*-*based concept tagging* To check, whether a term in the intermediate lexicon is a valid medical entry, we search each of the word in UMLS, a basic medical lexicon which associates each of the input word with its corresponding medical concept. The UMLS contains more than 1.5 million biomedical concepts and over 10 million associations between these concepts (Bodenreider 2004). We used Sense-Related module of Perl-based UMLS similarity package (McInnes et al. 2009) to measure semantic relatedness between input term and its associated concepts listed in UMLS. Exactly matched terms are identified along with their UMLS (Table 7). The resulting lexicon is named as: “*HL*-*f*”. Algorithm for concept tagging is given as follows:

**Table 7:** Sample list of words and their UMLS codes

S. no	Word	UMLS code
1	Sore throat	C0242429
2	Heart burn	C0018834
3	Stomach pain	C1963242
4	Abdominal cramps	C0000729
5	Dizzy	C0012833
6	Increased blood pressure	C2917273
7	Nausea	C0549206
8	Weak	C0004093
9	Moral distress	C1828099
10	Diarrhea	C0011991
11	Sleep walking	C0037672
12	Dysenteric diarrhea	C0277526
13	Weight gain adverse even	C2911647
14	Heart attack	C0027051
15	Heatstroke	C0018843
16	Silent migraine	C3203712

For example sentence, in sentence: “*The regular use of medicine given me relief in sore*-*throat and heart*-*burn*”, there are two words, namely “*sore*-*throat*” and “*heart*-*burn*”, which are tagged with UMLS concepts as follows: (1) C0242429 (sore-throat) and (2) C0018834 (heart-burn).

### Sentiment scoring

This module deals with the assignment of sentiment scores to each of the word in health-related sentiment lexicon using two options: (1) using SWN, and (2) using domain specific strategy.

#### SWN-based scoring

To assign sentiment scores to health-related opinion words, we use SentiWordNet (SWN), because of its wide coverage of words and their sentiment scores. The SWN is a general purpose lexicon with more than sixty thousand synsets obtained dynamically from WordNet (Asghar et al. 2014a, b).

Three numerical scores are associated to each of the synset. Each entry/word is assigned three sentiment scores: +ive, −ive, and neutral. The score ranges in the interval: 0.0–1.0, and the overall sum is equal to 1.0, for each of the word. Each entry in SWN takes the form:

2 $$W_{i} = \left\langle {p.o.s, swn.id,sen^{ + } , sen^{ - } ,sen^{o} , S, Gl} \right\rangle$$

where, p.o.s shows part-of-speech of the word, swn.id is the SWN key, $sen^{ + } ,\,sen^{ - } ,$ and $sen^{o}$ are the +ive, −ive, and neutral scores of $W_{i}$ such that $sen^{ + } + sen^{ - } + sen^{o} = 1$, S[vi] = {s0, s1, s3,…, sn} are the synsets of vi, and $Gl$ is the gloss description of $W_{i}$. Sample entries in the SWN lexicon are presented in Table 8.

**Table 8:** Sample SWN entries

POS	ID	Pos score	Neg score	Obj score	Synset terms	Gloss definition
Verb	02109404	0.5	0	0.5	tolerate#3	Have a tolerance for a poison or strong drug or pathogen or environmental condition; “The patient does not tolerate the anti-inflammatory drugs we gave him”
Adjective	02114746	0	0.5	0.5	infective#2 infectious#1	Caused by infection or capable of causing infection; “viruses and other infective agents”
Noun	14259133	0	0.625	0.375	temporal_arteritis#1	Inflammation of the temporal arteries; characterized by headaches and difficulty chewing and (sometimes) visual impairment
Adverb	00275035	0	0	1	asleep#1	Into a sleeping state; “he fell asleep”

POS represents part-of-speech, ID represents SWN-entry’s identification key; Pos Score, Neg Score, and Obj Score represent positive, negative and objective scores of entry respectively. Synset Terms represents synonyms set of the entry, and Gloss definition represents textual explanation of the entry

To evaluate correct sense of an opinion word having multiple senses, we consider three polarity scores: positive, negative, and objective of all senses of all of the parts-of-speech (P-O-S) of a word available in the SWN.

We compute three average values $Pol\_score^{ + } ,\,Pol\_score^{ - } ,$ and $Pol\_score^{o}$ for all of the senses of a word “${\text{w}}_{\text{i}}$” with respect to all parts-of-speech (POS):

3 $$Pol\_score^{ + } \left( {{\text{w}}_{\text{i}} } \right) = \frac{1}{\text{numSyn}}\mathop \sum \limits_{{{\text{i}} = 1}}^{\text{n}} pol^{ + } \left( {\text{i}} \right)$$

4 $$Pol\_score^{ - } \left( {{\text{w}}_{\text{i}} ,} \right) = \frac{1}{\text{numSyn}}\mathop \sum \limits_{{{\text{i}} = 1}}^{\text{n}} pol^{ - } \left( {\text{i}} \right)$$

5 $$Pol\_score^{o} \left( {{\text{w}}_{\text{i}} ,} \right) = \frac{1}{\text{numSyn}}\mathop \sum \limits_{{{\text{i}} = 1}}^{\text{n}} pol^{o} \left( {\text{i}} \right)$$

where, $Pol\_score^{ + } ,\,Pol\_score^{ - } ,$ and $Pol\_score^{o}$ represent the average sentiment score: +ive, −ive, objective of sense i for word ${\text{w}}_{\text{i}} ,$, and ${\text{numSyn}}$ is the sum of synsets of all possible P.O.S of the word ${\text{w}}_{\text{i}} .$

 For example the word “left” has ten entries in SWN, four senses for adjective category, five senses for noun, and one sense in adverb. The average positive, negative, and objective scores are computed as:

$$\begin{aligned} Pol_{score}^{ + } \left( w \right) & = \, \left( {0 + 0.125 + 0 + 0 + 0 + 0 + 0 + 0 + 0 + \, 0} \right) /10 = 0.125 /10 = {{0}}.{{0125}} \\ Pol_{score}^{ - } \left( w \right) & = \, \left( {0 + 0. \, 5 + 0 + 0 + 0 + 0 + 0 + 0 + 0 + 0} \right) /10 = 0. \, 5 /10 = {{0}}.{{05}} \\ Pol_{score}^{o} \left( w \right) & = \, \left( {1 + 0.375 + 1 + 1 + 1 + 1 + 1 + 1 + 1 + \, 1} \right)/10 = 9.375/10 = {{0}}.{{9375}} \\ \end{aligned}$$

Therefore, average positive, negative, and objective polarity scores for all senses of all P-O-S of the word “left” are 0.0125, 0.05, and 0.9375 respectively.

To compute final sentiment score of a word, we choose its maximum polarity “${\text{w}}_{\text{i}}$” as:

6 $$pol^{swn} \left( {{\text{w}}_{\text{i}} } \right) = \left\{ {\begin{array}{*{20}l} {pol^{ + } } \hfill &\quad {if\,max\left( {pol^{ + } , pol^{ - } , pol^{o} } \right) = pol^{ + } } \hfill \\ {pol^{ - } } \hfill & \quad{if\,max\left( {pol^{ + } , pol^{ - } , pol^{o} } \right) = pol^{ - } } \hfill \\ {pol^{o} } \hfill &\quad {else} \hfill \\ \end{array} } \right.$$

The $pol^{swn} \left( {{\text{w}}_{\text{i}} } \right)$ is +ive, if the average +ive score $\left( {pol^{ + } } \right)$ is higher than both average negative $\left( {pol^{ - } } \right)$ and objective $\left( {pol^{o} } \right)$ scores. We get the negative sentiment score by same principle. The sentiment score is objective, if average positive and average negative polarity scores are same or the average objective polarity score is greater than positive and negative. For example, the sentiment score triplet $\left\langle {pol^{ + } , pol^{ - } , pol^{o} } \right\rangle$ for a word “Left” is $\left\langle {0.0125, \, 0.05, \, 0.9375} \right\rangle$; therefore, $pol^{swn}$ (“Left”) = 0.9375. This word is objective. Same technique is used in the case of positive or negative polarity. The positive or negative words are presented along with their final dominant score and corresponding orientation.

#### Domain specific scoring

In health-related domain, most of the words have one sentiment class in SWN, whereas their occurrence in the annotated dataset indicates strong inclination with the other sentiment class. For example, the word “*tuberculin*” has objective sentiment score in SWN, but its occurrences in the +ive reviews are higher than the −ive class. Therefore, we change sentiment class and score of such domain specific words.

*Polarity class detection* In order to check frequency of a terms in a particular labeled class (i.e. +ive or −ive), we compute count-based probability (Bayes 2012) of each term in the testing dataset and its polarity class is predicted in the training dataset as follows:

7 $$polarity\_class\left( w \right) = \left\{ {\begin{array}{*{20}l} { + ive, } \hfill & \quad{ if \left( {\frac{{frequency\left( {w \in T_{ + } } \right)}}{{\left| {T_{ + } } \right|}}} \right) > \left( {\frac{{frequency\left( {w \in T_{ - } } \right)}}{{\left| {T_{ - } } \right|}}} \right) } \hfill \\ { - ive} \hfill &\quad {else} \hfill \\ \end{array} } \right.$$

where $\frac{{frequency\left( {w \in T_{ + } } \right)}}{{\left| {T_{ + } } \right|}}$ and $\frac{{frequency\left( {w \in T_{ - } } \right)}}{{\left| {T_{ - } } \right|}}$ are the probabilities of word **w** occurs in +ive and −ive reviews of training dataset set respectively, and T+ and T− are training datasets of +ive and −ive reviews respectively.

For example, the sentiment class of the word “*tuberculin*” is objective in SWN, whereas score of *prob*(*w*, *c*_*p*_) is higher than the *prob*(*w*, *c*_*n*_), showing that it has more tendency towards +ive class. A selected list of positive and negative words is presented in Table 9.

**Table 9:** Selected list of words and their polarity class

Word (uni/bi-gram)	SWN class	Polarity class detection using Eq. 7
Sore throat	Not found	−ive
Atheroma	Objective	−ive
Fibroelastosis	Not found	−ive
Tuberculin	Objective	+ive
Puberty	Objective	+ive
Heatstroke	Objective	−ive
Diarrhea	Objective	−ive
Heart burn	Not found	−ive

*Polarity score modification* When a word is either not found in SWN or its SWN-based polarity class (Eq. 6) is different from the predicted class (Eq. 7), we propose a modified polarity scoring method for the accurate scoring of such words. To calculate modified polarity score, we combine term frequency (tf), inverse document frequency (idf) and the count-based probability (Eq. 8). The proposed scoring scheme is an extension of the existing weighting method (Paltoglou and Thelwall 2010). They used weighted scoring mechanism and achieved satisfactory results in terms of accuracy. The major limitation of their approach is that they did not consider the importance of domain specific words, which results in accurate scoring of such words. To address this issue, we combine term frequency (tf), inverse document frequency (idf) and count-based probability (Eq. 8) as follows:

8 $$pol^{modified} = \left\{ {\begin{array}{*{20}l} {tf\,x\,idf\,x \left( {\frac{{frequency\left( {w \in T_{ + } } \right)}}{{\left| {T_{ + } } \right|}}} \right), } \hfill & {if \left( {\frac{{frequency\left( {w \in T_{ + } } \right)}}{{\left| {T_{ + } } \right|}}} \right) > \left( {\frac{{frequency\left( {w \in T_{ - } } \right)}}{{\left| {T_{ - } } \right|}}} \right) } \hfill \\ {tf\,x\,idf\,x \left( {\frac{{frequency\left( {w \in T_{ - } } \right)}}{{\left| {T_{ + } } \right|}}} \right),} \hfill & { if \left( {\frac{{frequency\left( {w \in T_{ + } } \right)}}{{\left| {T_{ + } } \right|}}} \right) < \left(\frac{{frequency\left( {w \in T_{ - } } \right)}}{{\left| {T_{ - } } \right|}}\right)} \hfill \\\end{array} } \right.$$

For example, the word “*Atheroma*” has objective polarity (1) in SWN, but using modified scoring technique (Eq. 8), the score of “*Atheroma*” becomes −2.8. Moreover, a bigram “*Heart burn*” is not found in SWN, and its score, using modified scoring technique becomes −1.6. A list of selected words is shown in Table 10.

**Table 10:** Selected list of words with modified polarity scores

S. no	Word (unigram/bigram)	UMLS code	SWN score	Modified score using Eq. 8
1	Sore throat	C0242429	Not found	Negative (1.2)
2	Heart burn	C0018834	Not found	Negative (1.6)
3	Stomach Pain	C1963242	Not found	Negative (1.9)
4	Abdominal cramps	C0000729	Not found	Negative (2.0)
5	Atheroma	C0264956	Neutral (1)	Negative (2.8)
6	Fibroelastosis	C0016038	Not found	Negative (1.7)
7	Trypanosomiasis	C0041227	Not found	Negative (2.1)
8	Tuberculin	C0022415	Neutral (1)	Positive (2.2)
9	Diarrhea	C0011991	Neutral (1)	Negative (1.7)
10	*Puberty*	C0034011	Neutral (1)	Positive (2)
11	Heatstroke	C0018843	Neutral (1)	Negative (2.5)
12	Silent migraine	C3203712	Neutral (1)	Negative (1.7)

## Experiments

To conduct different experiments, we used python-based libraries of Natural Language Toolkit (NLTK) (Bird et al. 2009) to implement all of the algorithms proposed for lexicon creation in the previous sections.

### Results

Precision, recall, F-score, and accuracy are the different metrics used to analyze the performance of the proposed system, computed as follows:

$$\begin{aligned} & Precision \left( p \right) = \frac{tp}{tp + fp} \\ & Recall \left( r \right) = \frac{TP}{tp + fn} \\ & F\text{-}measure = \frac{2\left( p \right)\left( r \right)}{p + r} \\ & {\text{Accuracy}} = \frac{tp + tn}{{{\text{tp }} + {\text{fp}} + {\text{tn}} + {\text{fn}}}} \\ \end{aligned}$$

where, tp, fp, tn, and fn represent the number of true +ive predictions, false +ive predictions, true −ive predictions and false −ive predictions respectively.

The first experiment investigates the effectiveness of polarity class detection measure $polarity\_class\left( w \right)$ computed in Eq. 7. Referring to the results shown in Table 10, almost all of the +ive and −ive polarity classes detected for the given words depict correct semantic orientation. The words “*Fibroelastosis*” and “*Tuberculin*” have close tendencies towards –ive and +ive class labels respectively. Because the former occurs in 25 −ive reviews and the latter is included in 19 +ive reviews. The majority of reviews on health refereeing to the word “*Diarrhea*” are −ive, therefore, our proposed measure (Eq. 7) correctly categorize it into −ive class. The uni-gram “*Puberty*” has neutral polarity in SWN, whereas it occurs mostly in +ive reviews (22 +ive and 2 −ive). Therefore, the word “*Puberty*” is placed in the +ive class. Therefore, our method reflects accurate polarity tendencies of words in health domain.

**Fig. 3:**
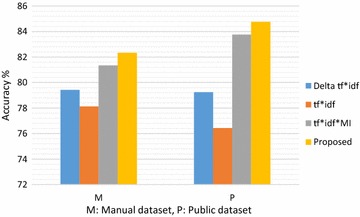
Accuracy-based performance evaluation of the proposed method

The next experiment aims at investigating the efficiency of the $tf {\text{x idf x }}\left( {\frac{{frequency\left( {w \in T_{ + } } \right)}}{{\left| {T_{ + } } \right|}}} \right)$ and $tf {\text{x idf x }}\left( {\frac{{frequency\left( {w \in T_{ - } } \right)}}{{\left| {T_{ + } } \right|}}} \right)$. We computed values of the baseline measures, namely: delta tf x idf, tf x idf and tf x idf x MI for each of the manual and public dataset and compared the result with the our proposed measure. Figure 3 depicts the accuracy-based evaluation of proposed measure on the given datasets. Our method performs better than the comparing methods. The accuracy of the proposed measure is about 3.4 %greater than that of tf x idf in given datasets. The performance of delta tf x idf is poor due to lack of word sense disambiguation. The tf x idf x MI performs better than the delta tf x idf in the given datasets.

Moreover, we observe that classification accuracies on public dataset are higher than those in manually compiled reviews. This is due the fact that publically available dataset is more refined in terms of low noise and has already used been used by research community in multiple experiments.

The next experiment aimed at investigating the practical usefulness of SentiHealth on the sentence sentiment classification task. For this purpose, we applied vote-switch algorithm (Lorraine et al. 2012) for computing the performing the sentiment classification of sentences into +ive, −ive, or neutral. The Algorithm 5 is used to evaluate the +ive and −ive words from the lexicon and the words having more votes are declared winner. The algorithm is applied at the review (document) level to a dataset of 26,060 users’ reviews. The review is labeled as +ive, if it has more positive sentences. We used the algorithm to classify the users’ reviews into +ive, −ive, or objective.

**Fig. 4:**
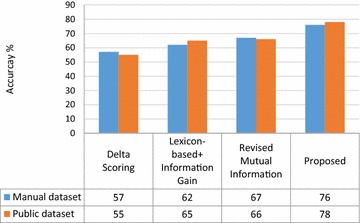
Lexicon-wise accuracy comparison with respect to vote-switch algorithm

We applied the vote-switch algorithm to evaluate the accuracy-based comparisons of proposed lexicon on manual and public dataset, as shown in Fig. 4. We observe that the SentiHealth (proposed) shows improved performance over the comparing lexicons, namely: Delta Scoring, Lexicon-based + Information Gain, and Revised Mutual Information. The improved results are due to enhanced sentiment detection and scoring of health-related domain specific words, which are not available in other lexicons. Overall, we observe that whatever the dataset is, the proposed (SentiHealth) gives best results. Our lexicon demonstrates improved results for both manual and public datasets over the comparing lexicons, which shows that it has better coverage of medical terms.

 Finally, we compare our hybrid approach for creating health-related sentiment lexicon with other related works. Table 11 summarizes the performance of different lexicons. There is only lexicon that implemented a hybrid approach that is different from our technique and used Information Gain to assign sentiment scores to health-related domain specific words (Goeuriot et al. 2012). In Asghar et al. (2015), the authors used supervised machine learning with same feature set as ours. The precision, recall and f-measure are low when we compare it with our technique. This may be due enhanced noise reduction that we implemented in our system. In another work (Demiroz et al. 2012), authors have used different technique with bag of words features and limited noise reduction. They concluded that supervised technique is more suitable than the unsupervised classifier. They used variation of term weighting measures for sentiment scoring of domain specific words. Moreover, their delta score updating method outperformed the comparing methods. However, experiments were performed on limited number of hotel and movie reviews, and therefore, the technique was not tested on medical terms to evaluate its effectiveness on health domain.

**Table 11:** Performance comparison with other methods

Work	Dataset	Noise Reduction	Features	Approach	Precision	Recall	F-measure
Goeuriot et al. (2012)	25,000 reviews	Filtering	Unigram and bigram	Hybrid (lexicon-based + information gain)	0.76	0.52	0.62
Asghar et al. (2015)	15,000 reviews	Filtering, tokenization, stop word removal, stemming	Unigram, bigram and trigram	Supervised (revised mutual information)	0.78	0.64	0.64
Demiroz et al.(2012)	9000 reviews	Filtering, stop word removal	Bag of words	Supervised (delta scoring)	0.75	0.48	0.58
Our work	26,060 reviews	Filtering, tokenization, stop word removal, lemmatization, spell correction, co-reference resolution	Unigram, bigram and trigram	Hybrid (boot strapping + corpus-based)	0.89	0.79	0.83

All the mentioned evaluations measures are as reported by their respective work

## Lexicon coverage

The proposed technique produced the sentiment lexicon providing wide coverage of health-related words. The lexicon captures 1520 words, 40 % +ive, 45 % −ive, and 15 % objective (neutral). Most of the words (78 %) stored in our proposed lexicon are not available in SWN. For example, Sore throat (−ive), heart burn (−ive), stomach pain (−ive) were not present in the existing general-purpose lexicon, namely SWN. About 75 % of the words, when compared to the proposed modified scoring scheme, have different polarity scores; a selected version of such terms is already presented in Table 10. The differences between the SWN and the proposed sentiment scores supports our supposition that creation of health-related sentiment lexicon is essentially required developing efficient sentiment analysis applications.

## Conclusions

This study addressed the problem of creating health-related sentiment lexicon by proposing a hybrid approach, which combines boot-strapping and corpus-based strategies. The proposed system consists of following modules: (1) data acquisition and pre-processing; (2)seed cache creation and lexicon expansion; (3) lexicon filtering and tagging; (4) polarity class detection; and (5) polarity score modification.

The proposed method assists in creating a sentiment lexicon from initial list of health-related seed words, expands it over different Web repositories, and fitters the irrelevant words by using co-reference PMI measure. An appropriate polarity class is decided for each of the word by proposing count-based probability measure. Moreover, accurate polarity score is assigned to words by introducing enhanced term weighting scheme. The results obtained in terms of different evaluation metrics, such as accuracy, precision, recall and F-measure depict the effectiveness of proposed method.

The limitations of the proposed method is that the expansion of seed cache needs be made over different medical lexicons, instead of web lexicons, which may result in a more comprehensive expansion of initial lexicon. The increased rate of irrelevant words is due to generalized nature of web repositories, more specific health-related lexicons should be exploited to reduce the noise in expanded lexicon. Another possible future direction is to investigate the dynamic updating of lexicon entries over different online repositories, such as web thesaurus and biomedical dictionaries. Analyzing the effect of multiple senses on sentiment classification of health-related words would be another extension of the current study.

## Abbreviations

SWN: SentiWordNet
UMLS: Unified Modeling Language System

## Footnotes

1 http://spellcheckplus.com.

2 http://freespellcheckers.com.

3 https://www.jspell.com/.

4 http://www.thesaurus.com/.
